# Spectral monitoring at SwissFEL using a high-resolution on-line hard X-ray single-shot spectrometer

**DOI:** 10.1107/S1600577521009619

**Published:** 2021-10-21

**Authors:** Christian David, Gediminas Seniutinas, Mikako Makita, Benedikt Rösner, Jens Rehanek, Petri Karvinen, Florian Löhl, Rafael Abela, Luc Patthey, Pavle Juranić

**Affiliations:** a Paul Scherrer Institut, Forschungsstrasse 111, 5232 Villigen, Switzerland; b European XFEL GmbH, Holzkoppel 4, 22869 Schenefeld, Germany; c Advanced Accelerator Technologies AG, 5234 Villigen, Switzerland; d Institute of Photonics, University of Eastern Finland (UEF), FI-80100 Joensuu, Finland

**Keywords:** free-electron laser, X-ray spectrometer, spectroscopy

## Abstract

The performance and parameters of the online photon single-shot spectrometer (PSSS) at the Aramis beamline of the SwissFEL free-electron laser are presented. The device operates between the photon energies 4 and 13 keV and uses diamond transmission gratings and bent Si crystals for spectral measurements on the first diffraction order of the beam. The device has an energy window of 0.7% of the median photon energy of the free-electron laser pulses and a spectral resolution (full width at half-maximum) Δ*E*/*E* on the order of 10^−5^. The device was characterized by comparing its performance with reference data from synchrotron sources, and a parametric study investigated other effects that could affect the reliability of the spectral information.

## Abstract 2

The characterization and commissioning of the X-ray photon single-shot spectrometer (PSSS) at the Aramis beamline of SwissFEL is presented. The device delivers online, non-invasive, high-resolution single-shot measurements of spectral profiles between 4 and 13 keV.

## 1. Introduction

The pulse-to-pulse measurement of spectra of self-amplified spontaneous emission (SASE) X-ray free-electron lasers (FELs) (Bergmann *et al.*, 2017 ▸) is of fundamental importance for several experimental techniques ranging from resonant inelastic X-ray scattering (RIXS) (Lemke *et al.*, 2013 ▸; Chergui, 2016 ▸; Obara *et al.*, 2017 ▸; Park *et al.*, 2019 ▸; Kimberg & Rohringer, 2016 ▸; Błachucki *et al.*, 2014 ▸; Kayser *et al.*, 2019 ▸) to protein crystallography (Tono *et al.*, 2015 ▸; Moreno-Chicano *et al.*, 2019 ▸). The SASE process changes the spectral properties of the X-ray pulse on a pulse-to-pulse basis, which requires a device for online, non-invasive measurements of the X-ray spectra for both experimental spectral normalization and performance optimization (Rehanek *et al.*, 2017 ▸). A good example of the use of an online spectrometer is in X-ray absorption spectroscopy (XAS), where measurements have been reported using both SASE and sample-transmitted spectra (Boutet & Hunter, 2018 ▸; Katayama *et al.*, 2013 ▸; Brenner *et al.*, 2019 ▸). Such spectrometers are also useful in high-pressure or high-energy density science, where scans have to be performed with a small number of shots, preventing monochromator scans (Harmand *et al.*, 2015 ▸). A large advantage in recording absorption spectra with the full SASE mode compared with the use of monochromatic light is that extreme intensity fluctuations are avoided, if the SASE spectrum does not contain the photon energy chosen by the monochromator (Boutet & Hunter, 2018 ▸). The SASE spectrum also carries useful information to set up FEL operational parameters and develop new operating modes (Rehanek *et al.*, 2017 ▸) and can be used to estimate pulse durations from the measurements of spectral spike widths (Malyzhenkov *et al.*, 2020 ▸; Huang *et al.*, 2017 ▸; Makita *et al.*, 2015 ▸).

Several different spectrometer designs have been used at FELs for these kinds of spectral characterizations (Inubushi *et al.*, 2012 ▸; Boesenberg *et al.*, 2017 ▸; Rich *et al.*, 2016 ▸; Svetina *et al.*, 2016 ▸; Makita *et al.*, 2015 ▸; Tono *et al.*, 2013 ▸). Experiments at the Linac Coherent Light Source (LCLS) used bent Si crystals with transmission gratings (Makita *et al.*, 2015 ▸). The bent Si crystals approach achieved high-resolution measurements (better than 0.2 eV at 8.3 keV), but were limited in spectral range and lost about half of the photon flux due to poor transmission (Zhu *et al.*, 2012 ▸) while the transmission gratings had a poorer resolution (1.2 eV at 6 keV) but much better transmission (Karvinen *et al.*, 2012 ▸). The SPring-8 Angstrom Compact Free Electron Laser (SACLA) used a transmission grating in combination with an elliptical mirror and a flat Si crystal to deliver online spectra with tunability in both resolution and spectral range (Katayama *et al.*, 2016 ▸). The Swiss-FEL photon single-shot spectrometer (PSSS) (Rehanek *et al.*, 2017 ▸) combines a transmission grating with bent Si crystals to create a spectrometer that has a good resolution, large spectral range, and good transmission for online spectral measurements of photon energies between 4 and 13 keV. Using this setup, the first order of the diffracted beam is used for spectral or intensity monitoring, while the zeroth order is transmitted downstream of the experiments.

This work presents the achievements and the characterized capabilities of the PSSS as a single-shot online X-ray spectrometer. We demonstrate that the PSSS can deliver a full width at half-maximum (FWHM) resolution of Δ*E*/*E* ≃ 5 × 10^−5^ and a spectral window of up to 0.7% of the photon energy over the working range of the device.

## 2. Setup

**Figure 1:**
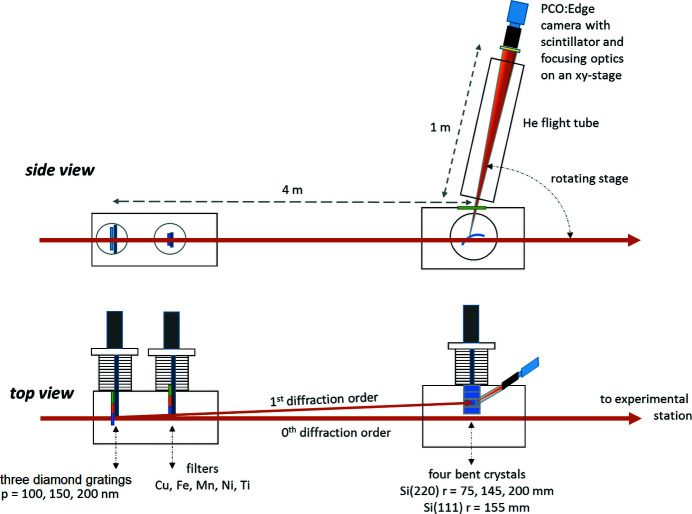
Setup of the PSSS in SwissFEL (side view and top view, compare Rehanek *et al.*, 2017 ▸). The diamond gratings are located 4 m upstream and diffract the beam in the horizontal plane. The first diffracted order is then Bragg reflected from a bent Si crystal and projected to a camera that can be rotated in a distance of 1 m around analyser crystals, providing a θ/2θ operation scheme. A helium-filled flight tube between the crystal and the camera ensures sufficient transmission even at lower photon energies.

The working principle of the PSSS is shown in Fig. 1 ▸. The diamond grating diffracts the incoming FEL beam in the horizontal plane, sending the first order to the bent crystal spectrometer while the zeroth order continues further downstream with 80% or more of the incoming flux. Monitoring the spectra online in this fashion reduces the heat load on the spectrometer optics (Boesenberg *et al.*, 2017 ▸). The first order is Bragg-reflected from the bent Si crystal in the spectrometer and projected onto a detector, as shown in Fig. 1 ▸. The detector is a PCO:Edge 5.5 camera with an objective that is focused onto a Ce:YAG scintillator. The diffraction gratings have pitches of 100 nm, 150 nm and 200 nm. The grating pitches are chosen such that the first-order diffracted beam is always far enough from the zeroth order so that the crystals can be put safely into it and do not block or affect the propagation of the main beam to the experimental station downstream. The first-order efficiency can be enhanced by tilting the diamond gratings up to 60°. For the bent Si crystals, three Si(220) crystals with bending radii of 75 mm, 145 mm and 200 mm, and one Si(111) crystal with a bending radius of 155 mm can be chosen. All of the Si crystals are 10 µm thick. More information about the PSSS construction are given by Rehanek *et al.* (2017 ▸) and Juranić *et al.* (2018 ▸).

**Figure 2:**
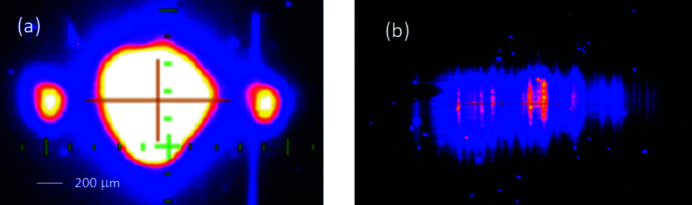
Diffracted orders of the full beam observed by the saturated profile monitors between the grating and Bragg crystal chambers (*a*), and the raw image of the spectrum on the PSSS detector (*b*).

Additional profile monitors situated both before and after the Bragg crystal chambers allow for the destructive observation of the diffracted beam and to see the Bragg diffraction in transmission. These monitors are Ce:YAG scintillators with a camera/lens unit (Juranić *et al.*, 2018 ▸). Fig. 2 ▸(*a*) shows the diffracted beam on the profile monitor, while Fig. 2 ▸(*b*) shows the raw image of the spectrum on the PCO.Edge camera of the spectrometer. The RMS size of the photon beam in the horizontal and vertical directions at the PSSS ranges from about 150 to 400 µm, depending on the photon energy and operating mode.

**Figure 3:**
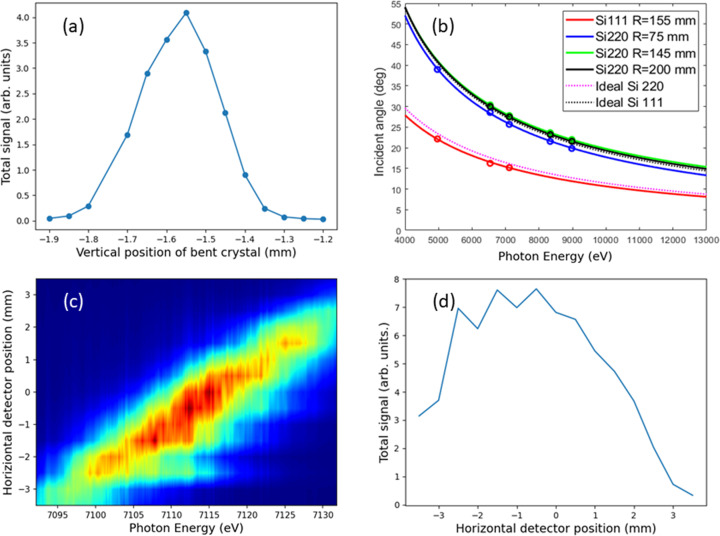
Vertical position scan for a 12 keV beam of the Si bent crystals on the first-order diffraction (*a*), the Bragg angles from various *K*-shell foil edges (circles) compared with ideal Bragg reflection curves for each crystal (*b*), and the detector response tests as a function of detector position perpendicular to the Bragg reflection, showing the average spectra (*c*) and integrated intensity and pulse energy (*d*) at a photon energy of about 7.1 keV and pulse energy of about 200 µJ.

The PSSS setup requires precise alignment of the Si bent crystals to the beam. The vertical position of the crystal is the most important parameter to maximize the signal once the correct Bragg angles were selected for a chosen energy window. The setup is typically conducted in the non-invasive mode: the diamond gratings upstream diffract the beam, and the analyser crystals are placed in the first diffraction order. An exemplary measurement, conducted at 12 keV photon energy and with a pulse energy of approximately 110 µJ, is shown in Fig. 3 ▸(*a*).

The precise energy calibration of the spectrometer was performed by inserting several filter foils into the beam and identifying the transmission edges. By fitting the observed position on the camera to the expected Bragg angles, the geometry, miscut of the crystals and mounting tolerances were taken into account. The used foils were Ti (*K*-edge 4.966 keV, 10 µm thickness), Mn (*K*-edge 6.54 keV, 20 µm thickness), Fe (*K*-edge 7.11 keV, 10 µm thickness), Ni (*K*-edge 8.33 keV, 12.5 µm thickness) and Cu (*K*-edge 8.98 keV, 20 µm thickness). The photon energy of the FEL was set to the absorption edges of the foils, and the Bragg angle of the PSSS was scanned to find the motor positions that matched the photon energies of the absorption edges for all crystals. The measured points were then used to fit a theoretical Bragg curve with an additional free parameter to take the miscut of the crystals into account. These fits were then used to create a look-up table for all relevant motor positions across the energy window for each crystal. The resulting points and Bragg curves are shown in Fig. 3 ▸(*b*). Note that we also show the Bragg curves without miscuts for Si(111) and Si(220) (dashed lines).

The sensitivity across the detector was investigated by scanning the detector position perpendicular to the Bragg reflection and comparing the integrated intensity against the simultaneous measurements taken with the online gas-based pulse energy monitor (Juranić *et al.*, 2018 ▸). The field of view of the detector is about 4 mm, so the spectra becomes cut off as one approaches that limit, and the integrated intensity drops off as a part of the spectrum is cut out, as shown in Figs. 3 ▸(*c*) and 3(*d*).

## 3. PSSS performance and discussion

### 3.1. Operational parameters

#### 3.1.1. Si crystals and transmission gratings

The first step of the PSSS commissioning process was to determine the performance of different Si crystals and transmission gratings. From the Bragg angles [Fig. 3 ▸(*b*)], we can see the reachable energy windows for the different silicon crystals. The Si(220) reflection can be used over the complete range of the PSSS (4 to 13 keV), while the Si(111) reflections can be used from 4 keV to 8 keV due to the limited range of the detector rotation stage due to other beamline components, limiting us to Bragg reflections from 14° to 60°.

**Figure 4:**
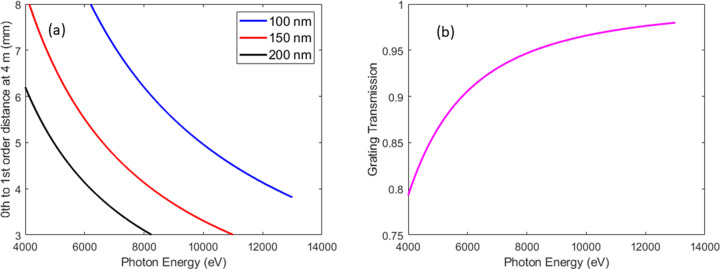
Separation from the zeroth- to first-order (mm) beam at the bent crystals chamber from diffraction by different pitches of the diamond gratings (*a*), zeroth-order transmission of the diamond gratings as a function of photon energy (*b*).

Fig. 4 ▸(*a*) shows the dependence of the separation of the zeroth- and first-order beams as a function of photon energy for the three available diamond gratings with 100, 150 and 200 nm pitches. The region between 14° and 60° is where non-invasive operation of the PSSS is possible, corresponding to between 3 mm and 8 mm beam separation. Fig. 4 ▸(*b*) shows the calculated transmission of the diamond gratings as a function of photon energy, calculated with the Henke tables (Henke *et al.*, 1993 ▸; http://henke.lbl.gov/optical_constants/tgrat2.html). The transmitted beam has a transmission between 80% at 4 keV and 98% at 13 keV photon energy. The transmission efficiency of the gratings has been measured and reported elsewhere (Juranic *et al.*, 2019 ▸).

#### 3.1.2. Si crystal alignment, energy calibration and detector sensitivity

The average integrated intensity of the spectra on the detector shows a very strong dependence on the vertical position of the bent Si crystal in the beam, as shown in Fig. 3 ▸(*a*). The integrated intensity drops by about 50% for a 110 µm displacement of the crystal for an optimum position, highlighting the sensitivity of the device to misalignments and shifts in the beam position.

The difference between the measured Bragg angle values and those expected from the calculations are explained by the miscuts in the manufacturing process of the Si crystals (Rehanek *et al.*, 2017 ▸). The offset between the measured crystal angles and the ideal Bragg angles is obvious in Fig. 3 ▸(*b*), where the three different Si(220) crystals have slightly different Bragg curves, offset from each other by a constant. The angular offsets of the Bragg angle for the four crystals were determined from these fits, and are shown in Table 1 ▸.

Fig. 3 ▸(*c*) shows the average spectra plotted as a function of the sensor position perpendicular to the Bragg reflection. The sensitivity seems to be homogeneous over the scanned region, though the spectra, and the integrated intensity, start being clipped and reduced as the spectrum is moved out of the sensor’s field of view. Fig. 3 ▸(*d*) indicates that the best sensor position for the spectral center to minimize this clipping is between −2.5 mm and 1 mm, giving about a 3.5 mm effective field of view to reliably observe the spectrum.

#### 3.1.3. FEL beam profile

Previous work has noted that the spectral intensity distribution can depend on the part of the FEL beam profile that is being Bragg reflected by the crystals (Makita *et al.*, 2015 ▸; Rehanek *et al.*, 2017 ▸). The homogeneity of the beam profile across the sampled portion of the beam being Bragg reflected should be as good as possible to ensure good spectral intensity measurements.

**Figure 5:**
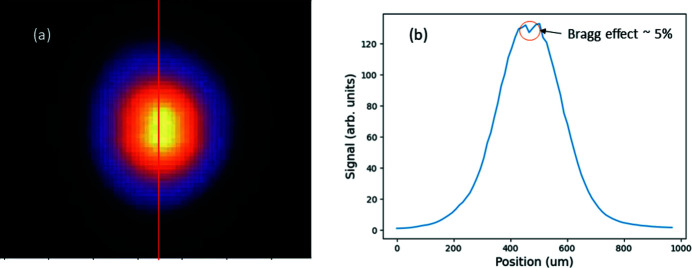
(*a*) Color map of the FEL beam profile when the bent crystals are in the zeroth-order beam and attenuated to prevent saturation effects. (*b*) The projection of the pixels along the red line in (*a*) that shows the loss of signal due to the Bragg reflection in transmission, accounting for about 5% of the intensity at the maximum.

Fig. 5 ▸(*a*) shows the profile of the transmitted beam with a Bragg crystal positioned on the main beam (without the use of a diamond grating) that was acquired with a profile monitor downstream of the Bragg crystals (attenuated to avoid saturation). The diffracted portion of the beam is revealed by the small intensity drop in the beam center. The amount of the Bragg reflection is estimated as the maximum difference between the sides and the Bragg dip in the middle of the profile, as shown in Fig. 5 ▸(*b*). The dip in signal due to the Bragg reflection is about 5% near the maximum. From the tiny amount of the diffraction and the uniformity of the transmitted beam profile, we anticipate that the influence of the intensity on the measured spectral distribution of the beam is negligible as long as the Bragg crystal is centered.

#### 3.1.4. Measured energy resolution and spectral range

**Figure 6:**
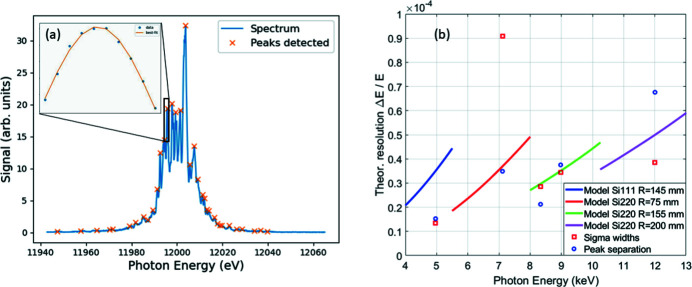
(*a*) A spectrum used for peak finding and Gaussian curve fitting taken at 12 keV. (*b*) Resolution measurements of the PSSS at the filter foil *K*-edges and at 12 keV compared with theoretical expectations.

Fig. 6 ▸ shows the expected PSSS resolution at several different photon energies. The measurements were taken with the PSSS non-invasive mode, looking at the first order of the transmission grating. There are two measures to determine the resolution of a spectrometer: the minimum spike width that it can resolve, and the minimum peak separation that is still visible. The evaluation for these two values was performed using the Python Scipy library which evaluated the peaks whose prominence was at least ten times above the standard deviation of the noise in the background signal at the edges of the spectrum. The smallest separation between these peaks was considered as the best resolution observed. The peaks were then fitted with a Gaussian model (including an offset) to find their widths. Those peaks that had the offset near the background level of the spectrum were compared, and the smallest sigma values of these peaks was taken as the best sigma width resolution. A thousand spectra were evaluated to find the smallest sigma values and peak separations for each photon energy. Fig. 6 ▸ shows the minimum spike width and peak separation that were observed at 4.966 keV, 7.114 keV, 8.333 keV, 8.996 keV and 12.015 keV photon energies and compares them with the expected values. They are also compared with each other and expected theoretical values in Table 2 ▸, which were evaluated using the expected beam size for that photon energy.

Note that the measurements for the minimum spike width are a convolution of the resolution of the spectrometer and the natural spike width of the FEL spectrum, which likely limits the measurable energy resolution.

The sigma and peak separation measurements are specifically close to the expected energy resolution for the Si(220) crystal with *R* = 155 mm, and reasonably close to the crystal with *R* = 200 mm. It is possible that the photon spectrum at this energy was not as optimized as the other ones, causing the spectrum itself to have broader spikes. However, the resolution of the spectrometer stays in the 10^−5^ Δ*E*/*E* resolution range for all measurements, matching the expectations set during the device design.

The spectral range of the PSSS matched the theoretical estimates (Rehanek *et al.*, 2017 ▸), ranging between 0.56% and 0.74% with the correct selection of Bragg crystals and bending radii. Si(220) crystals with bending radii of 200 mm, 145 mm and 75 mm are used for ranges of 5.5–8 keV, 8–10.5 keV and 10.5–13 keV, respectively. A Si(111) crystal with a bending radius of 155 mm is used to provide a similar spectral range for photon energies between 4 and 5.5 keV. The range is large enough to clearly visualize the standard mode spectrum and its tails, as seen in Fig. 6 ▸.

### 3.2. Experience with the PSSS

**Figure 7:**
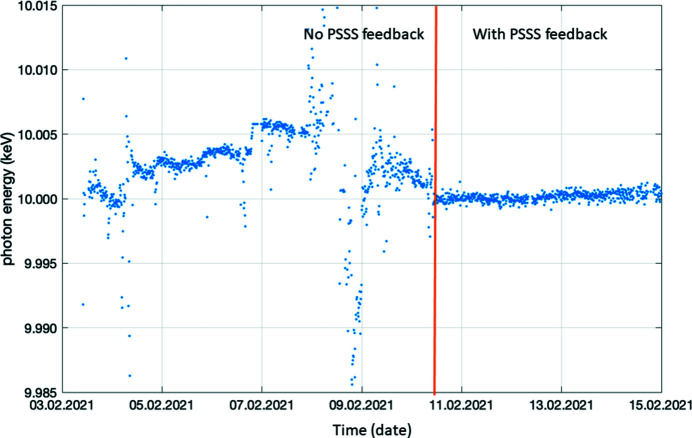
The spectral center of mass evaluated from the PSSS before (left of orange line) and after (right of orange line) the PSSS photon energy feedback was enabled on the machine during a week of operation.

The PSSS went into full operation for users and machine operators after the implementation of a user-friendly graphical user interface for spectral measurements based on look-up tables created from the calibration data. To further help with the optimization of the FEL spectral settings, a fast algorithm was developed to evaluate the center of energy and the FWHM of a Gaussian fit to the smoothed spectrum on a shot-to-shot basis. Further tools created by the accelerator scientists use this data for feedbacks for the machine, and the PSSS is being used regularly to correct drifts and instabilities of the mean photon energy and spectral width via a feedback system and an optimizer to the SwissFEL accelerator settings. The PSSS has been used even when the end-stations use monochromators for an experiment, as the PSSS spectral bandwidth evaluation can be used to optimize the smallest bandwidth the FEL can achieve so that the maximum amount of photons possible pass through a monochromator’s bandpass, increasing the signal at the experiment. A good example of such an optimization feedback, and its effect on stabilizing the photon energy, is shown in Fig. 7 ▸. Overall, the device has been extremely helpful for machine optimization and experimental work.

## 4. Conclusions

The PSSS enables the online monitoring of shot-to-shot fluctuations in spectral properties of the FEL, delivering a spectral range of up to 0.76% of the photon energy, and a resolution of bandwidths Δ*E*/*E* < 5 × 10^−5^, consistent with the theoretical estimates. Using Fe, Ni and Cu *K*-edges from XAS spectra from reference data from synchrotrons, the spectrometer was calibrated to be capable of visualizing the FEL spectrum in the standard mode while preserving high-resolution visualization of individual spikes. The PSSS enables the operators and users of the Aramis beamline to monitor and optimize the performance of the FEL and the experiment online, and is a crucial component of the SwissFEL diagnostics suite.

## Figures and Tables

**Table 1:** Bragg angle offsets due to miscuts for the four bent crystals

Crystal	Offset (°)
Si(111), *R* = 155 mm	−0.217
Si(220), *R* = 75 mm	−0.898
Si(220), *R* = 145 mm	1.149
Si(220), *R* = 200 mm	0.537

**Table 2:** Expected theoretical and measured resolutions by photon energy; the measured resolutions were evaluated using peak separations and by looking at the smallest peak widths found

Photon energy (eV)	Expected resolution (eV)	Peak separation (eV)	Sigma width (eV)
4966	0.174	0.076	0.066
7112	0.263	0.248	0.646
8333	0.250	0.177	0.237
8996	0.315	0.337	0.309
12015	0.610	0.816	0.462
